# Evaluation of Myocardial Protection in Prolonged Aortic Cross-Clamp Times: Del Nido and HTK Cardioplegia in Adult Cardiac Surgery

**DOI:** 10.3390/medicina61081420

**Published:** 2025-08-06

**Authors:** Murat Yücel, Emre Demir Benli, Kemal Eşref Erdoğan, Muhammet Fethi Sağlam, Gökay Deniz, Hakan Çomaklı, Emrah Uğuz

**Affiliations:** 1 Department of Cardiovascular Surgery, Ankara Bilkent City Hospital, Ankara 06800, Turkey; benemredemir@gmail.com (E.D.B.); kemal_esref@hotmail.com (K.E.E.); dr.m.fethisaglam@gmail.com (M.F.S.); dr.gokaydeniz@gmail.com (G.D.); hakan.comakli@gmail.com (H.Ç.); emrahuguz@gmail.com (E.U.); 2 Department of Cardiovascular Surgery, Ankara Yıldırım Beyazıt University Faculty of Medicine, Ankara 06010, Turkey

**Keywords:** cardioplegic solutions, myocardial protection, aortic cross-clamping, adult cardiac surgery

## Abstract

*Background and Objectives:* Effective myocardial protection is essential for successful cardiac surgery outcomes, especially in complex and prolonged procedures. To this end, Del Nido (DN) and histidine-tryptophan-ketoglutarate (HTK) cardioplegia solutions are widely used; however, their comparative efficacy in adult surgeries with prolonged aortic cross-clamp (ACC) times remains unclear. This study aimed to compare the efficacy and safety of DN and HTK for myocardial protection during prolonged ACC times in adult cardiac surgery and to define clinically relevant thresholds. *Materials and Methods:* This retrospective study included a total of 320 adult patients who underwent cardiac surgery under cardiopulmonary bypass (CPB) with an aortic cross-clamp time ≥ 90 min. Data were collected from the medical records of elective adult cardiac surgery cases performed at a single center between 2019 and 2025. Patients were categorized into two groups based on the type of cardioplegia received: Del Nido (*n* = 160) and HTK (*n* = 160). The groups were compared using 1:1 propensity score matching. Clinical and biochemical outcomes—including troponin I (TnI), CK-MB, lactate levels, incidence of low cardiac output syndrome (LCOS), and need for mechanical circulatory support—were analyzed between the two cardioplegia groups. Subgroup analyses were performed according to ACC duration (90–120, 120–150, 150–180 and >180 min). The predictive threshold of ACC duration for each complication was determined by ROC analysis, followed by the analysis of independent predictors of each endpoint by multivariate logistic regression. *Results:* Intraoperative cardioplegia volume and transfusion requirements were lower in the DN group (*p* < 0.05). HTK was associated with lower TnI levels and less intra-aortic balloon pump (IABP) requirement at ACC times exceeding 180 min. Markers of myocardial injury were lower in patients with an ACC duration of 120–150 min in favor of HTK. The propensity for ventricular fibrillation after ACC was significantly lower in the DN group. Significantly lower postoperative sodium levels were observed in the HTK group. Prolonged ACC duration was an independent risk factor for LCOS (odds ratio [OR]: 1.023, *p* < 0.001), VIS > 15 (OR, 1.015; *p* < 0.001), IABP requirement (OR: 1.020, *p* = 0.002), and early mortality (OR: 1.016, *p* = 0.048). Postoperative ejection fraction (EF), troponin I, and CK-MB levels were associated with the development of LCOS and a VIS > 15. Furthermore, according to ROC analysis, HTK cardioplegia was able to tolerate ACC for up to a longer duration in terms of certain complications, suggesting a higher physiological tolerance to ischemia. *Conclusions:* ACC duration is a strong predictor of major adverse outcomes in adult cardiac surgeries. Although DN cardioplegia is effective and economically advantageous for shorter procedures, HTK may provide superior myocardial protection in operations with long ACC duration. This study supports the need to individualize cardioplegia choice according to ACC duration. Further prospective studies are needed to establish standard dosing protocols and to optimize cardioplegia selection according to surgical duration and complexity.

## 1. Introduction

Optimal myocardial protection is one of the cornerstones of surgical success, particularly in cardiac surgery with cardiopulmonary bypass (CPB) and aortic cross-clamp (ACC). Inadequate protection during this critical period may result in ischemia–reperfusion injury, postoperative low cardiac output syndrome (LCOS), arrhythmias, and increased mortality [1]. This protection is typically provided by cardioplegic solutions during CPB. These solutions decrease metabolic activity and increase ischemic tolerance. The efficacy of myocardial protection is directly related not only to the surgical technique but also to the type, composition, and administration strategy of the cardioplegia solution used.

The prolonged duration of ACC in cardiac surgery remains a critical prognostic indicator as it increases the risk of ischemia–reperfusion injury and subsequent complications [1]. Conventional cold-blood cardioplegics complicate the surgical workflow with the need for frequent repeat doses because of their short duration of action [2]. Del Nido (DN) and histidine-tryptophan-ketoglutarate (HTK) are two cardioplegia solutions that are widely used in modern adult cardiac surgery, especially in cases with long ACC duration, can be administered as a single dose, and do not interrupt the surgical flow [3]. DN, developed for pediatric surgery, is an extracellular solution containing lidocaine and mannitol and is known for its cell membrane stabilization and osmotic protective effects [4]. In recent years, it has become widespread in adult surgery and an effective and cost-effective option, especially for ischemia durations of up to 90–120 min [5]. DN provides effective myocardial protection for up to 90 min without the need for re-dosing, especially in short- and medium-term procedures, and is cost-effective [6]. HTK, on the other hand, is an intracellular solution with high buffering capacity owing to its histidine content and provides metabolic support with ketoglutarate. Although more expensive, they provide longer protection (usually exceeding two hours) and are used in cases requiring long ACC periods [7,8]. Both DN and HTK are considered safe for myocardial protection; however, the available literature is limited. DN offers the advantage of reducing intraoperative interruptions due to its simple dosing and efficacy, while HTK provides superior protection in longer procedures [9]. Although both cardioplegia solutions are widely used in clinical practice, direct studies on the comparative efficacy of DN and HTK solutions in adult cardiac surgery are limited, particularly for ACC times exceeding 150–180 min. Most studies in the literature have insufficient statistical power and tend to focus on smaller and heterogeneous patient groups or address one or a few specific parameters of myocardial protection [10,11]. Furthermore, subgroup analyses according to the duration of ischemia, one of the key factors determining cardioplegia efficacy, have generally not been performed [9]. There is also no consensus in the literature regarding administration protocols, dosing intervals, or biochemical/clinical evaluation criteria [10]. However, the lack of advanced comparative analyses examining the differential effects of DN and HTK on major clinical outcomes, such as mortality, low cardiac output syndrome (LCOS), and high inotrope requirement, makes it difficult to develop clinical decision algorithms in this field, and the choice of cardioplegia is often based on individual surgeon experience or institutional habits [9,10].

This study aimed to compare the effects of DN and HTK, two different cardioplegia solutions used in adult cardiac surgery, on postoperative clinical outcomes. Biochemical markers (Tn I, CK-MB, and lactate) and clinical outcomes (EF, LCOS, VIS, and arrhythmias) were analyzed together in both cardioplegia groups. In addition, subgroup analyses were performed over time periods defined according to the ACC duration. Thus, the protection profile provided by both cardioplegic solutions at different ischemia durations was revealed multidimensionally. In addition, the predictive value of DN and HTK cardioplegia solutions on major postoperative complications (LCOS, VIS > 15, IABP requirement, and mortality) associated with ACC duration was determined, and the differences in clinical tolerance of both solutions to this duration were revealed. For this purpose, advanced statistical analysis techniques (e.g., ROC analysis, cut-off determination, and multivariate regression) were used to develop the efficacy of cardioplegia in a clinical decision support model.

## 2. Materials and Methods

### 2.1. Study Design and Ethical Approval

This single-center, retrospective, matched cohort study was designed to compare the efficacy and safety of DN and HTK cardioplegia solutions in adult cardiac surgery patients with long ACC times. The study protocol was approved by the institutional Ethics Committee (Approval No: E2-23-3790). This study was conducted in accordance with the principles of the Declaration of Helsinki. Informed consent was not obtained due to the retrospective design of this study.

### 2.2. Patient Selection

**Figure 1:**
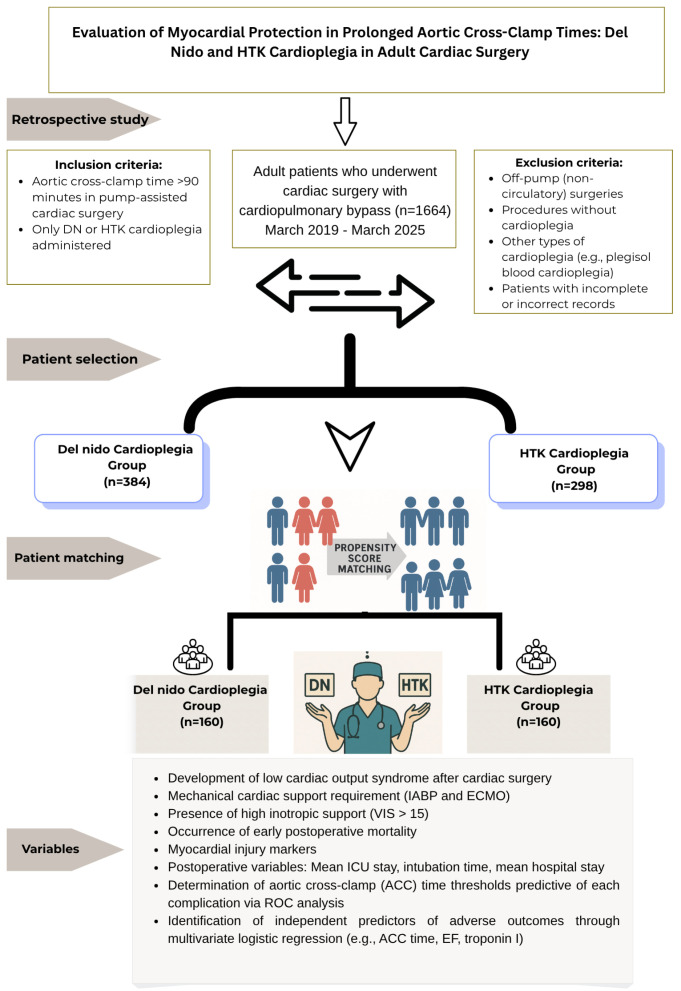
Flowchart of the study design, patient selection, group allocation, and outcome measures in the comparative evaluation of Del Nido and HTK cardioplegia in adult cardiac surgery.

All patients who underwent cardiac surgery under CPB between March 2019 and March 2025 at our institution were systematically screened using an electronic patient record system. All surgeries and clinical follow-up data included in this study were completed and finalized as of April 2025. Only patients with fully available 30-day outcome data were included in the final analysis. During the screening process, surgical records, surgical procedure codes (ICD-10), surgical notes, perfusionist records, and anesthesia records were used as primary data sources. Initially, the patient pool was identified using institutional surgical databases via automatic electronic querying, without manual intervention or subjective filtering. While the initial cohort was identified through automated electronic screening without manual intervention, the subsequent propensity score matching (PSM) was conducted using clinically relevant covariates—including age, sex, BMI, ejection fraction (EF), and surgical type—selected based on expert consensus and supported by the prior literature. First, using the method described above, a total of 1664 patients who underwent cardiac surgery under CPB were identified through electronic querying. Predefined inclusion and exclusion criteria were applied to this pool. Inclusion criteria were age ≥18 years, intraoperative DN or HTK cardioplegia application, and ACC duration ≥90 min. The ≥90 min threshold was selected to specifically include patients exposed to prolonged ischemia. This value is commonly used in the literature to define prolonged ACC duration and has been associated with increased risk of myocardial injury and postoperative complications in adult cardiac surgery [12,13]. Off-pump surgeries were excluded to ensure comparability of myocardial protection and biochemical parameters. However, the use of cardioplegic solutions other than DN or HTK and patients with missing data were excluded from this study. Patients with missing data on key clinical variables, including demographic information, ACC duration, cardioplegia type, postoperative troponin I or CK-MB levels, and outcome measures (e.g., LCOS, IABP, mortality), were excluded from the analysis. In line with these criteria, 682 patients (384 in the DN group and 298 in the HTK group) were included in the analysis. Owing to the retrospective and non-randomized nature of this study, there was a risk of selection bias, especially when the choice of cardioplegia was based on surgical complexity. Propensity score matching (PSM) was applied to reduce this bias, increase comparability between groups, and reduce imbalances in key demographic and clinical variables. In the matching process, a 1:1 nearest-neighbor matching method was preferred, and the caliper width was set to 0.2 standard deviations (SD). Matching was performed using a multivariate logistic regression model including age, sex, BMI, preoperative EF, and type of surgery (isolated valve surgery, isolated coronary artery bypass grafting, and combined surgeries). After matching, 160 patients from each group were included in the analysis. The balance between the groups was confirmed using the standardized mean difference (SMD < 0.1). The primary outcome measures of this study were postoperative troponin I and CK-MB levels (at 4 and 24 h), LCOS incidence, high inotropic requirement (VIS > 15), IABP use, and early (30-day) mortality. Patient selection criteria, group classification based on cardioplegia type, and the overall study design flowchart are summarized in Figure 1.

### 2.3. Myocardial Protection

The type of cardioplegia was determined according to the institution’s standard practice, considering the expected ischemic duration, type of surgical procedure, and anatomical factors. In practice, HTK is generally preferred in long-duration or complex cases, whereas DN is preferred in short-duration operations.

DN cardioplegia was prepared with a 1:4 crystalloid-blood dilution and administered at a dose of 20 mL/kg for 5 min at a temperature of 4–8 °C and pressure of 80–100 mmHg. In cases with an ACC duration exceeding 90 min, half a dose was administered every 60 min. The route of administration is usually antegrade but retrograde in cases of aortic insufficiency or left ventricular hypertrophy. HTK cardioplegia: A commercially prepared solution was administered at a dose of 20 mL/kg over 7–8 min, at a temperature of 4–8 °C and pressure of 80–100 mmHg. In cases with an ACC duration exceeding 120 min, a half dose was administered again. The administration was generally antegrade and supported by a retrograde route if necessary.

### 2.4. Data Collection and Definitions

All data for patients who met the inclusion criteria were obtained from the Hospital Information Management System (HIMS). No preliminary protocol was used in this study. To ensure methodological transparency and reporting integrity, this study was structured and reported in accordance with the PRISMA-ScR (Preferred Reporting Items for Systematic Reviews and Meta-Analyses Extension for Scoping Reviews) guideline [14]. All biochemical measurements were performed in accordance with the institution’s standardized laboratory protocols and obtained from data electronically registered in the HIS system. TnI and CK-MB levels were measured at two time points postoperatively: 4 h after ICU admission and 24 h after surgery. In addition, sodium, potassium, creatinine, hematocrit, and lactate levels were measured preoperatively and 6 h after ACC. Postoperative outcomes were monitored until hospital discharge and 30 days postoperatively.

The primary endpoints of this study were mortality and biochemical myocardial damage, assessed using postoperative TnI and CK-MB levels. Measurements were taken at 4 h and 24 h postoperatively.

**Figure 2:**
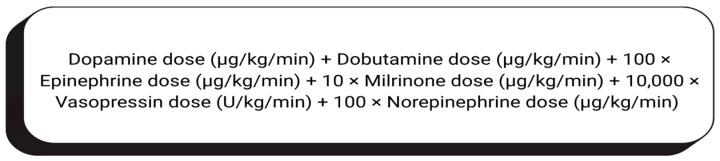
VIS (vasoactive–inotropic score) formula.

Secondary endpoints:

- Preoperative and postoperative ejection fraction (EF) change.
- Low cardiac output syndrome (LCOS) is defined as a systolic blood pressure (SBP) < 90 mmHg or cardiac index < 2.0 L/min/m^2^ + need for inotropic or mechanical support [15].
- The vasoactive inotrope score (VIS) was calculated using a standard formula based on adrenaline, noradrenaline, dobutamine, milrinone, and vasopressin doses. The VIS formulation is illustrated in Figure 2. Consistent with adult cardiac surgery studies in the literature, a VIS > 15 was considered a high inotropic support requirement [16].
- Need for mechanical support (IABP, ECMO).
- Postoperative arrhythmias (AF, VF).
- Intubation time, intensive care unit (ICU) stay, and length of hospital stay were determined.

Major complications evaluated within 30 days postoperatively included acute kidney injury, liver dysfunction, cerebrovascular accident (stroke), multiple organ dysfunction syndrome (MODS), reoperation, reintubation, rehospitalization, deep sternal infection, postoperative atrial fibrillation (AF), low cardiac output syndrome (LCOS), pneumonia, and mortality. All complications were retrospectively evaluated according to standardized clinical diagnostic criteria. Acute kidney injury was defined as a ≥1.5-fold increase in serum creatinine level compared to the preoperative value or the need for postoperative dialysis [17]; acute liver injury was defined as new signs of liver failure or a total bilirubin level > 3 mg/dL (approximately 51 µmol/L) [18]; stroke was defined as a new focal neurologic deficit and imaging findings supporting this condition; and mortality was defined as in-hospital deaths occurring within 30 days postoperatively. Other complications were defined in accordance with the current clinical guidelines and diagnostic criteria and were verified from patient files and recorded.

### 2.5. Statistical Analysis

Data were summarized as means, standard deviations, medians, frequencies, and percentages. Categorical variables are expressed as numbers and percentages, while continuous variables are reported as mean ± SD or median. Data distribution was evaluated using the Shapiro–Wilk and Kolmogorov–Smirnov tests. For group comparisons, an independent sample *t*-test was used for normally distributed variables, and the Mann–Whitney U test was used for non-normally distributed data. Categorical data were analyzed using chi-square or Fisher’s exact tests, and paired data were analyzed using McNemar’s test. To reduce baseline imbalances between groups, propensity score matching with a 1:1 nearest-neighbor algorithm without redundancy was applied, and the caliper width was set as 0.2 SD. Variable balance after matching was evaluated according to SMD < 0.1 criterion. The magnitude of the difference between the groups in continuous variables was calculated using Cohen’s d, and the effect size was classified as small (≥0.2), medium (≥0.5), or large (≥0.8). Subgroup analysis according to ACC duration was performed for four groups as 90–120, 120–150, 150–180, and >180 min. The VIS was analyzed both as a continuous variable and as a categorical variable (high/low), using a threshold of >15.

The Statistical Package for the Social Sciences (SPSS) version 27.0 was used for statistical analysis. GraphPad Prism v 10.1.0(316) for Windows 64-bit was used for graphical figures, Microsoft^®^ Excel^®^ MSO for Microsoft 365 (Version 2503 Build 16.0.18623.20116) and 64-bit for tables, Microsoft^®^ Visio^®^ 2019 MSO (Version 2505 Build 16.0.18827.20102) 64-bit for graphs and images, and Canva Pro for flowcharts.

## 3. Results

### 3.1. Preoperative Characteristics

Initially, PSM was performed using the following variables: age, sex, body mass index (BMI), preoperative EF, and type of surgery, to reduce imbalances between the groups before and after PSM. The pre- and post-PSM information for the listed variables is shown in Table 1. Detailed demographic and clinical characteristics of the matched cohort (*n* = 160 DN, *n* = 160 HTK) included in the analysis after PSM are presented in Table 2. The mean age was 55.01 ± 11.82 years in the DN group and 56.7 ± 9.6 years in the HTK group, with no statistically significant difference between the groups (*p* = 0.211). The sex distribution was similarly balanced between the groups (female: 58.75% vs. 49.03%, *p* = 0.261). There were no significant differences in body mass index (BMI) (*p* = 0.425) or type of surgery (*p* > 0.05 (Table 1)).

In addition, analysis of the following was conducted: hypertension (HT) (16.88% vs. 18.75%, *p* = 0.661), diabetes (DM) (12.50% vs. 13.75%, *p* = 0.741), hyperlipidemia (HL) (21.88% vs. 18.13%, *p* = 0.402), peripheral arterial disease (PAD) (10.63% vs. 13.13%, *p* = 0.489), chronic obstructive pulmonary disease (COPD) (8.13% vs. 6.25%, *p* = 0.516), active smoking (11.88% vs. 15.63%, *p* = 0.516) and preoperative EF (49.5 ± 7.5% vs. 48.5 ± 8.1%, *p* = 0.186).

### 3.2. Perioperative and Procedural Features

The perioperative and procedural data for both groups are presented in Table 3. When the DN and HTK cardioplegia groups were compared, significant differences were observed in the perioperative and procedural parameters. The mean total CPB time was 196.04 ± 30.97 min in the DN group and 201.86 ± 38.42 min in the HTK group. This difference was not statistically significant (*p* = 0.060). The mean aortic clamping time (ACC) was 145.8 ± 33.26 min in the DN group and 150.64 ± 35.99 min in the HTK group, and this difference was not significant (*p* = 0.317). The mean intraoperative temperature was 30.69 ± 5.59 °C in the DN group and 30.12 ± 1.77 °C in the HTK group (*p* = 0.140). However, there was a significant difference in the volume of cardioplegic solution administered. While the mean volume administered in the DN group was 1028.75 ± 322.02 mL, this value was significantly higher in the HTK group and was measured as 1417.81 ± 362.40 mL (*p* < 0.001). On the other hand, the initial dose of cardioplegia (DN and HTK 1250.63 + 254.77 and 1695.63 ± 328.58, respectively) and total cardioplegia doses were significantly higher in the HTK group (2039.38 + 528.48 vs. 1722.19 ± 317.45 in DN and HTK, respectively). The need for defibrillation was less in the DN group (3.75% vs. 9.68%, *p* = 0.042), while the need for temporary pacing was similar between the groups (1.25% vs. 2.58%, *p* = 0.388). Cardioplegia administration was performed by antegrade only (AC) or in combination with both antegrade and retrograde (ARC) routes. There was no significant difference in the route of cardioplegia administration between the groups (*p* = 0.312 and *p* = 0.312, respectively).

### 3.3. Early Postoperative Period and Clinical Outcomes

Table 4 compares the clinical effects of DN and HTK cardioplegia solutions in the early postoperative period. The mean duration of the intensive care unit (ICU) stay was 39.9 ± 23.79 h in the DN group and 39.75 ± 23.04 h in the HTK group, and no statistically significant difference was observed between the groups (*p* = 0.976). The mean duration of intubation was 10.7 ± 9.30 h in the DN group and 11.24 ± 12.30 h in the HTK group. There was no significant difference in this variable between the groups (*p* = 0.654). The mean length of hospital stay was 8.51 ± 2.32 days in the DN group and 9.09 ± 2.99 days in the HTK group, and the difference did not reach statistical significance (*p* = 0.182). When the postoperative drainage amounts were analyzed, it was found to be 684.38 ± 240.03 mL in the DN group and 661.25 ± 240.28 mL in the HTK group. There was no statistically significant difference between the two groups in this parameter (*p* = 0.319). In terms of blood products used, HTK patients required significantly more erythrocyte transfusions than DN patients (*p* < 0.05).

### 3.4. Pharmacologic and Mechanical Cardiac Support

Postoperative inotropic and mechanical support data are presented in Table 4. Although HTK patients required higher dobutamine doses, the inotrope score indicating cumulative inotrope support was calculated as 14.13 ± 10.79 on average, in the DN group, while this value was 12.86 ± 10.87 in the HTK group. No significant difference was found between the two groups in this parameter (*p* = 0.189). When the need for mechanical cardiac support was analyzed, the rates of intra-aortic balloon pump (IABP) use were 8.13% and 6.88% in the DN and HTK groups, respectively (*p* = 0.671). The extracorporeal membrane oxygenation (ECMO) requirement was 1.25% in the DN group and 3.13% in the HTK group, and this difference was not statistically significant (*p* = 0.252). Early postoperative complications, including renal injury and mortality rates, did not differ significantly between the groups (*p* > 0.05). In contrast, rehospitalization rates, LCOS, and postoperative AF rates were similar in both groups (*p* > 0.05) (Table 4).

### 3.5. Biochemical and Myocardial Damage Markers

The mean postoperative sodium levels were 135.51 ± 5.16 mg/dL in the DN group and 134.02 ± 3.80 mg/dL in the HTK group. A statistically significant difference was found between the two groups in terms of postoperative sodium levels (*p* = 0.003) (Table 5).

Creatine kinase-MB (CK-MB) levels measured in the first 4 h postoperatively were 29.82 ± 9.41 ng/mL in the DN group and 29.62 ± 8.66 ng/mL in the HTK group. CK-MB levels measured at the 24th postoperative hour were 38.85 ± 16.61 ng/mL in the DN group and 39.80 ± 17.61 ng/mL in the HTK group, respectively. No statistically significant differences were observed between groups at either time point (Table 5).

The mean TnI levels measured at the 4th postoperative hour were 6.55 ± 4.10 ng/mL in the DN group and 5.58 ± 2.21 ng/mL in the HTK group, and a significant difference was found between the groups at this time point (*p* = 0.003). However, there was no statistically significant difference in Tn I levels measured at the 24th postoperative hour (*p* = 0.151) (Table 5).

**Figure 3:**
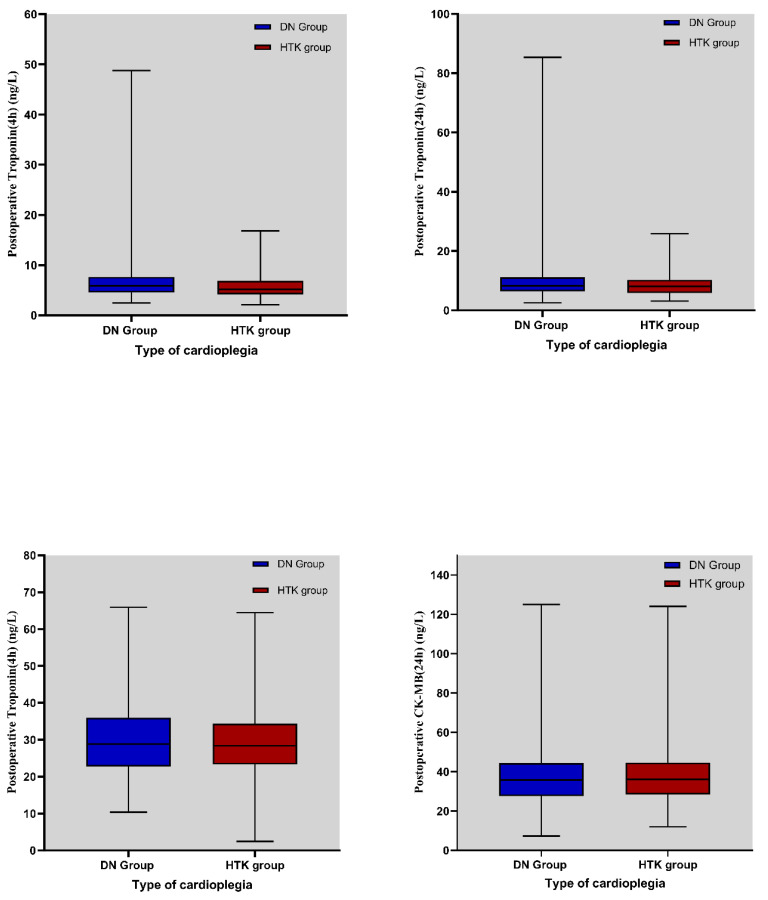
Distribution of CK-MB and Tn I (TnI) values in the first 4 h and first 24 h postoperatively in DN cardioplegia and HTK cardioplegia groups.

The distribution of CK-MB and Tn I values in the DN cardioplegia and HTK cardioplegia groups in the first 4 and 24 h postoperatively is shown in Figure 3.

### 3.6. Subgroup Analysis According to ACC Duration

To assess myocardial protection, patients were categorized according to ACC duration (90–120 min, 120–150 min, 150–180 min, and >180 min). When the ACC duration was ≥150 min, postoperative lactate levels were higher in the DN group. When ACC exceeded 180 min, Tn I levels were significantly higher at 4 h in DN patients, but this difference was reduced at 24 h. In addition, IABP utilization was significantly lower in patients with HTK (Table 6).

In subgroup analyses based on surgical type, no significant differences were observed between DN and HTK in the isolated CABG (*n* = 59) and aortic surgery (*n* = 67) groups in terms of postoperative EF, cardiac biomarkers, LCOS, VIS > 15, or IABP use (all *p* > 0.05). Although HTK was associated with numerically lower troponin I and CK-MB levels in these subgroups, the differences were not statistically significant. However, in the combined valve + CABG group (*n* = 50), early troponin I levels at 4 h were significantly lower in the HTK group (5.23 ± 1.80 vs. 7.22 ± 2.69 ng/mL, *p* = 0.0038), suggesting superior early myocardial protection in this high-risk cohort.

**Figure 4:**
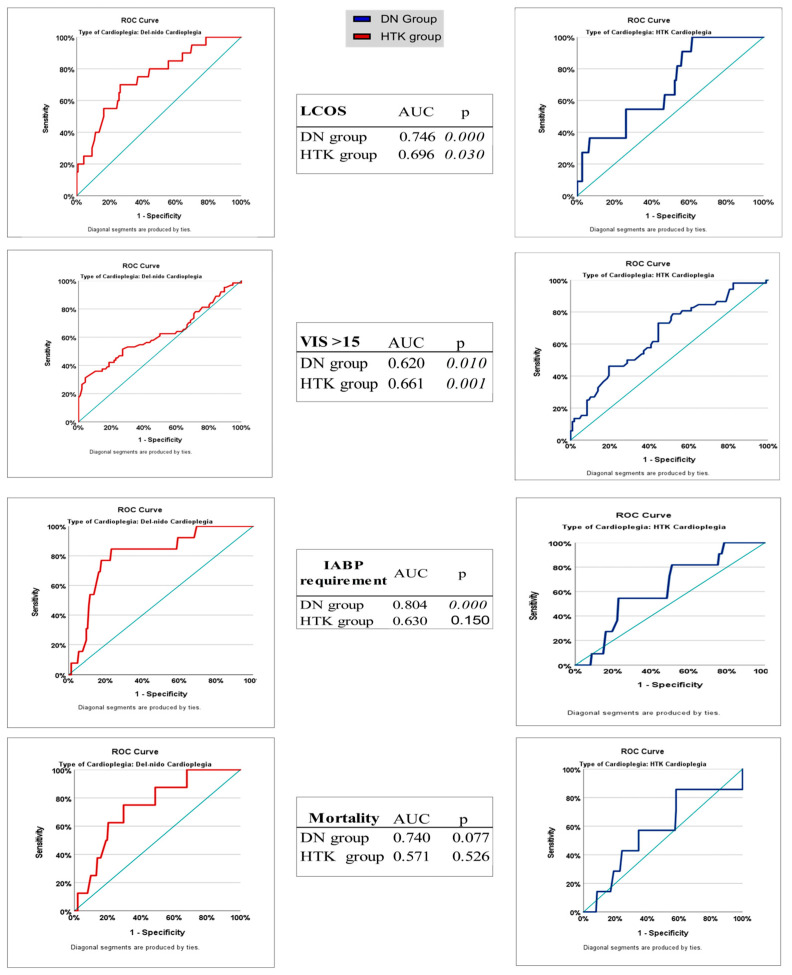
Diagnostic performance of aortic cross-clamp duration for predicting postoperative complications in Del Nido versus HTK cardioplegia: ROC curve analysis.

ROC analyses were performed to evaluate the association between ACC duration and major postoperative outcomes (development of LCOS, high inotrope requirement [VIS > 15], mortality, and IABP requirement) in the DN and HTK cardioplegia groups, and to identify potential cutoff values of these durations in predicting related complications (Table 7). In the DN group, ACC duration was a significant discriminator in predicting the development of LCOS (AUC, 0.746; 95% CI: 0.633–0.860; *p* = 0.000), with a cut-off value of 161 min, a sensitivity of 70%, and a specificity of 73.6%. For the same outcome, it showed a poor predictive value, with an AUC of 0.696 in the HTK group. This difference was not statistically significant (*p* = 0.074). The DN group also showed the highest diagnostic accuracy in predicting the need for IABP, with an AUC of 0.804 (*p* = 0.000), and the cut-off value for this outcome was 168.5 min. In the HTK group, the AUC for the same parameter was 0.630, which was not statistically significant (*p* = 0.150). For an outcome VIS > 15, the AUC was 0.661, and sensitivity was higher in the HTK group (73.1%, *p* = 0.001), while the specificity was higher in the DN group (95.8%), but sensitivity was limited (31.3%). For mortality, the AUC in the DN group was relatively high (0.740); however, the difference was not statistically significant (*p* = 0.077), whereas, in the HTK group, the AUC was only 0.571 (*p* = 0.526). These findings are supported by Figure 4, which includes ROC curve comparisons.

Multivariate logistic regression analyses were performed to determine independent predictors of major postoperative complications in patients exposed to prolonged ACC in the DN and HTK cardioplegia groups (Table 8).

In terms of LCOS development, age (OR: 0.960; 95% CI: 0.924–0.998; *p* = 0.041), prolonged ACC duration (OR: 1.023; 95% CI: 1.010–1.036; *p* < 0.001), low postoperative EF (OR: 0.917; 95% CI: 0.865–0.971; *p* = 0.003), and high troponin I level 24 h after surgery (OR, 1.154; 95% CI: 1.053–1.265; *p* = 0.002) were found to be significant independent risk factors. ACC duration (OR, 1.015; 95% CI: 1.008–1.022; *p* < 0.001) and low postoperative EF (OR, 0.965; 95% CI: 0.936–0.995; *p* = 0.021) were significant predictors of the need for high inotrope levels (VIS > 15). Although cardioplegia type was included in the multivariate analysis, it did not reach statistical significance as a predictor. In terms of IABP requirement, only prolonged ACC duration (OR: 1.020; 95% CI: 1.008–1.032; *p* = 0.002) and elevated CK-MB level at 24 h (OR, 1.027; 95% CI: 1.007–1.049; *p* = 0.010) were statistically significant; other variables did not reach significance. In terms of early postoperative mortality, high BMI (OR: 0.866; 95% CI: 0.750–1.001; *p* = 0.049) and long ACC duration (OR: 1.016; 95% CI: 1.000–1.031; *p* = 0.048) were independent significant predictors. Overall, prolonged ACC duration was the strongest and most consistent predictor of all four clinical endpoints. In addition, a low postoperative ejection fraction and biomarkers of myocardial injury (troponin I and CK-MB) contributed significantly to some outcomes.

## 4. Discussion

Effective myocardial protection is crucial in cardiac surgery, especially in complex and prolonged procedures, to reduce ischemia–reperfusion injury and improve patient outcome. DN and HTK cardioplegia solutions offer different benefits and limitations, depending on the complexity and duration of cardiac surgery. This study compared DN and HTK multifaceted in adult cardiac surgeries with long ACC times, focusing on their efficacy and safety profiles under prolonged ischemic conditions. Our aim was to contribute to clinical decision making by providing clearer insights into the comparative performance of these two solutions under conditions of myocardial ischemia.

There is a paucity of studies in the literature that directly compare the myocardial protective performance of DN and HTK at different ACC durations. Many existing studies are limited to small, heterogeneous patient cohorts or focus on only a few specific parameters of myocardial protection [10,11]. This study provided a multidimensional profile of the protective efficacy of both solutions over varying ischemic durations by performing subgroup analyses based on ACC timeframes. ROC-based analyses identified ACC time thresholds associated with the development of complications in DN and HTK patients. In our findings, DN cardioplegia was characterized by a volume advantage and lower transfusion requirement when the operation time was <150 min, whereas HTK provided biochemically better myocardial protection in operations lasting ≥180 min. The concept of “gray zone” for ACC duration of 120–150 min was defined and converted into a recommendation.

The mechanism of action of DN cardioplegia involves a sodium channel blockade via lidocaine. It requires re-dosing approximately every 60 min. Currently, the simple administration protocol has made DN a practical, effective, and economical option in cases with moderate ischemic durations. The need for hemodilution and associated transfusion is also reduced because of its lower-volume administration [19,20]. In contrast, the requirement for large-volume administration of HTK can lead to significant hemodilution and often requires postoperative transfusions to ensure hemodynamic stability [20]. In this study, DN cardioplegia showed advantages in reducing intraoperative cardioplegia volume and lowering transfusion requirements compared with HTK, possibly because of the smaller required dose volume and reduced hemodilution effect, in agreement with the literature. On the other hand, more hyponatremia was observed in the HTK group than in the DN group due to its low sodium formulation. However, hyponatremia is clinically mild. None of the patients developed symptomatic hyponatremia, and no neurological complications were reported. These results suggest that the adverse effects associated with HTK, although statistically significant, did not lead to clinically significant outcomes. In this study, although routine ultrafiltration was not systematically performed during CPB, fluid management strategies, such as ultrafiltration, were used in patients who required it. However, the use of ultrafiltration techniques to control intraoperative fluid balance and reduce volume overload and electrolyte disturbances that may occur with large volumes of crystalloid solutions, such as HTK, may alleviate these effects and reduce dependence on transfusions.

In our study, the incidence of ventricular fibrillation (VF) after ACC was higher in the HTK group than in the DN group. This finding is consistent with trends reported in the literature [21,22]. The VF episodes observed in this study were transient and effectively controlled using standard defibrillation protocols. Furthermore, these arrhythmias were not associated with adverse clinical outcomes such as increased inotrope requirement, LCOS development, or prolonged intensive care unit stay. The lower incidence of VF in the DN group after ACC may be explained by the protective effects of lidocaine, magnesium, and mannitol on myocardial cell membrane stability and ion regulation [23,24]. These components reduce the risk of VF after reperfusion by stabilizing myocardial cell membranes and intracellular ion flow [25]. In addition to its antiarrhythmic effects, lidocaine has cytoprotective effects, such as anti-apoptotic and anti-hemolytic properties [26]. This feature of DN cardioplegia may have contributed to the lower VF rate. Although DN is associated with a lower incidence of ventricular fibrillation, VF is inherently multifactorial. It is influenced by factors such as preoperative conduction abnormalities, electrolyte imbalances, ischemia duration, and intraoperative myocardial manipulation. Therefore, these findings should be cautiously interpreted. Future studies should evaluate preoperative ECG findings, intraoperative electrolyte changes, and temperature changes during reperfusion to develop a deeper understanding of the risk of VF.

Troponin I (TnI) and CK-MB levels are critical markers of myocardial injury and provide insight into the efficacy of cardioplegia [27]. In the literature, no significant difference was observed in TnI and CK-MB levels between the DN and HTK cardioplegia groups at ACC times of 120–150 min, representing a moderate ischemic period [28]. In this study, the myocardial protection profiles of DN and HTK were similar at this time interval. No significant differences were observed in terms of clinical outcomes. However, this does not mean that the groups are equivalent. However, the limited sample size and lack of statistical power should be taken into consideration. Although effect size analyses (Cohen’s d < 0.2) are supportive of non-inferiority by indicating clinically small differences, the ACC duration range of 120–150 min may be considered a “gray zone” that should be specifically examined in prospective studies with larger samples and homogeneous surgical procedures. In this critical interval, the decision between DN and HTK may enter the realm of *clinical equilibrium* or *therapeutic uncertainty*, where no single solution is unequivocally superior to others for all patients. In clinical practice, this highlights the need to individualize the choice of cardioplegia in cases in this gray area according to patient characteristics and surgical predictions. For example, in patients with sensitivity to electrolyte balance or the risk of repeat aortic clamping, the flexibility of DN redosability may be advantageous. In contrast, if the operation can be completed with a single dose of cardioplegia, HTK may be a more appropriate option. Therefore, this interval stands out as a decision point where individualized cardioprotective strategies can be developed, considering the unique advantages of both cardioplegia solutions.

In this study, both TnI levels and IABP utilization rates were significantly lower in the HTK cardioplegia group than in the DN group in patients with ACC duration exceeding 180 min. These findings suggest that HTK may provide more effective myocardial protection than DN during prolonged ischemic periods. This superiority may be related not only to the high buffering capacity of HTK but also to its synergistic components such as alpha-ketoglutarate (promoting ATP production) and tryptophan (membrane stabilization and reduction in oxidative stress) [29]. Furthermore, HTK has the potential to prevent ischemia–reperfusion injury by reducing intracellular edema and calcium overload owing to its low sodium and calcium contents [30]. The possibility of a single-dose administration does not interrupt the surgical flow and avoids the cumulative dilutional effects that can be seen in DN. These physiological and operational advantages make HTK a more effective cardioprotective agent in procedures exceeding 180 min. However, although higher troponin I levels at 4 h in the DN group suggest the possibility of biochemically increased myocardial damage, this difference was not reflected in clinical outcomes, such as postoperative EF, LCOS, need for mechanical support, or mortality. This biomarker-clinical outcome divergence reflects the uncertainties encountered in the clinical interpretation of the troponin levels. TnI elevation may be related to cardioplegia efficacy, reperfusion properties, myocardial edema, or transient metabolic stress. Furthermore, short-term troponin elevation may not represent permanent myocardial damage unless left ventricular function is impaired. Furthermore, the fundamental biochemical differences between DN and HTK cardioplegia solutions—DN being extracellular and HTK being intracellular—lead to unique effects on protective mechanisms [9]. These pathophysiological differences may explain the changes in troponin kinetics and myocardial responses at different durations.

Other important indicators of myocardial protection efficacy are EF and inotropic support requirements [31]. Both cardioplegia groups experienced postoperative decreases in EF, but subgroup analysis showed a more pronounced decrease in EF in the DN group at ACC times > 180 min. These findings are consistent with those of previous studies suggesting that HTK may contribute to myocardial stability under prolonged ischemic conditions [32]. In contrast, some animal model studies have found that DN cardioplegia better preserved diastolic and systolic left ventricular function than HTK, but these differences usually disappeared after reperfusion [33].

The vasoactive–inotropic score (VIS) is an important parameter indicating the need for postoperative cardiovascular support [34]. In this study, although the dobutamine doses were higher in the HTK group, the total VISs were similar between the two groups. This apparent discrepancy is likely due to differences in clinical preference or patient-specific hemodynamic goals and does not directly indicate myocardial dysfunction. VIS is a scoring system based on the total effect of multiple doses of inotropic and vasoactive agents (dopamine, adrenaline, noradrenaline, and milrinone). Therefore, a higher dose of a single agent may not significantly change the total VIS if the dose of other agents is low. The higher use of dobutamine in the HTK group may have been due to the need for right ventricular support, particularly in multivalvular and complex surgeries. This suggests that composite scoring systems, such as the VIS, may mask drug-based details and individualized treatment strategies.

Serum lactate levels are another reliable and indirect measure of metabolic stress and ischemia–reperfusion injury [35]. Elevated serum lactate levels after cardiac surgery reflect impaired tissue perfusion and an increased anaerobic metabolism. Excluding hypovolemia, elevated lactate levels after surgery are associated with low cardiac output. Studies have reported that DN cardioplegia results in lower serum lactate levels, possibly because of its formulation that stabilizes cellular metabolism and limits anaerobic processes [35]. Although HTK enables prolonged cardiac arrest, intracellular effects of prolonged ischemia may increase serum lactate levels. In our study, while the total ACC durations did not reveal statistically significant lactate differences, we observed an increase in lactate levels for DN beyond 150 min of ACC, suggesting that lactate trends should be carefully considered when using DN for prolonged ischemic durations.

In clinical studies, a higher incidence of VF was observed in the HTK group [21]. In our study, a higher incidence of VF was observed in the HTK group than in the DN group after ACC. Therefore, this situation should be clinically considered. However, these episodes are usually transient and controlled using standard defibrillation protocols. Furthermore, this was not associated with adverse clinical outcomes, such as higher inotrope requirement, development of LCOS, or prolonged intensive care unit stay.

In a subgroup analysis of patients who underwent isolated CABG, HTK cardioplegia was associated with lower postoperative TnI and CK-MB levels. On the other hand, while parameters such as LCOS and IABP requirement were statistically similar, the HTK group consistently showed lower injury markers, indicating that HTK exhibits a favorable profile in surgeries more prone to ischemia, such as combined CABG.

Receiver operating characteristic (ROC) analysis is a powerful method for evaluating the predictability of postoperative complications with certain clinical parameters. In our study, we sought to answer the question “When does the risk of complications become apparent if I use which agent?” by estimating the ACC duration using ROC analysis. In the current literature, no direct study has compared the cut-off thresholds that can predict postoperative complications of DN and HTK cardioplegia types with ROC analysis over ACC duration. Willekes and Duan et al. compared the clinical outcomes of DN and HTK with ACC durations of 90–150 min and longer, but ROC-based cut-offs and sensitivity-specificity analyses were not discriminatively presented in these studies [9,36]. Similarly, although there are observations on the safe use of DN as a single dose for a dosing duration of more than 90 min, these studies did not include an ACC duration cut-off analysis [6]. In this study, for the first time, ROC-based cut-off values were determined for important postoperative outcomes such as LCOS, VIS > 15, mortality, and IABP according to ACC duration in the DN and HTK groups, and the statistical power (AUC, sensitivity, specificity) of operational ACC tolerance thresholds as the threshold duration of both cardioplegics was measured. In our study, ROC analyses of ACC durations showed high diagnostic accuracy in predicting outcomes such as LCOS and IABP requirement in the DN group (AUC: 0.746 for LCOS; cut-off: 161 min; AUC: 0.804 for IABP; cut-off: 168.5 min). In contrast, although these cutoff times were longer in the HTK group, the AUC values were low and did not reach statistical significance. Similarly, in terms of the need for high inotropic support, such as VIS > 15, the HTK group had a higher sensitivity of 73.1% (the rate of catching patients who will develop the need for inotropes when using HTK), while the DN group had a higher specificity of 95.8% (the power to say that if VIS > 15 did not develop in patients with DN, there were no complications). These findings suggest that DN may more accurately exclude the risk of complications, whereas HTK is more sensitive in terms of early diagnosis. However, both groups showed limited diagnostic accuracy in terms of mortality prediction; the AUC was clinically remarkable at 0.740 only in the DN group, but statistical significance was not achieved. These results suggest that the cut-off values determined according to the duration of ACC do not reflect the ischemia tolerance limit of cardioplegia but rather the threshold at which the risk of complications becomes apparent. Thus, a higher cut-off value does not necessarily imply better ischemia tolerance; this value should always be evaluated in combination with diagnostic accuracy parameters such as AUC, sensitivity, and specificity. The predictive power of DN cardioplegia with higher AUC values, especially for outcomes such as LCOS and the need for IABP, suggests that the clinical predictability and confidence interval of this strategy are more consistent. Furthermore, the ability of HTK to demonstrate a higher physiological tolerance to ischemia and to tolerate longer ACC times before the onset of these complications provides an important guide for complex cases in which prolonged clamp times are anticipated. Conversely, the lower intraoperative cardioplegia volume and reduced need for transfusion with DN provide compelling arguments for its use in less complex, shorter procedures, where minimizing exposure to blood products and optimizing resource utilization are priorities.

In light of this information, for clinical practice, DN cardioplegia may be preferred for ACC duration < 150 min because of its proven efficacy, lower intraoperative volume, less transfusion requirement, and favorable effect on VF after ACC. For ACC duration > 180 min, HTK cardioplegia is recommended because of its better biochemical stability, lower TnI levels, and reduced need for IABP during prolonged ischemic periods. Its higher physiological tolerance to ischemia makes it a more robust choice for highly complex and lengthy procedures. In the ACC 120–150 min range, the choice of solution should be based on operational foresight, dosing flexibility, and patient-specific risk factors. The ACC 150–180 min is an area of treatment uncertainty. For this specific period, further research is needed to refine the recommendations, taking into account the findings of this study.

## 5. Limitations

This study had some limitations. First, the type of cardioplegia was not randomized but was determined according to criteria such as type of surgery, ischemic duration, and complexity of the procedure in accordance with local institutional protocols. Although the inherent biases of retrospective designs are mitigated by PSM, they cannot be eliminated. As this may lead to a potential selection bias, the PSM method was applied. Second, owing to the retrospective nature of this study, the findings may not be generalizable to all populations or institutions. The findings should be interpreted at a correlational level, and direct causal relationships should be avoided. Third, although this study was conducted at a single center and clinical management protocols were largely standardized, the involvement of different surgical teams did not completely eliminate the potential for team-based variation in practice. Furthermore, the patient population was heterogeneous, potentially affecting the statistical power of comparing the levels of myocardial protection for the two cardioplegia solutions. Fourth, the assessment of myocardial injury was limited to early (4 and 24 h) biomarkers such as Tn I, CK-MB, and lactate. Long-term outcomes such as cardiac function, EF change, and development of heart failure were not evaluated. Despite these limitations, the present study provides practical guidance for clinical decision making in adult cardiac surgery. By identifying differential patterns of efficacy between Del Nido and HTK cardioplegia across various ischemic durations and procedural complexities, our findings may assist clinicians in tailoring myocardial protection strategies to individual patient profiles. This personalized approach may contribute to optimizing early outcomes, particularly in surgeries involving prolonged aortic cross-clamp times.

## 6. Conclusions

An important observation that emerged from our study is that, consistent with the available literature, standardized dosing and administration protocols for both DN and HTK cardioplegia in adult cardiac surgery are still lacking. Despite their widespread use, differences in re-dosing intervals, administration techniques, and evaluation criteria for myocardial protection make comparisons in the literature difficult and lead to uncertainties in clinical decision-making processes. Our findings suggest that, instead of a uniform approach to cardioplegia selection, an individualized strategy should be adopted based on surgical complexity, expected ischemic duration, and institutional capabilities. Based on our findings, we recommend a pragmatic approach to the clinical decision-making algorithm for cardioplegia selection in adult cardiac surgery. In cases with an ACC duration < 150 min, Del Nido cardioplegia appears to be preferable because of its lower cardioplegia volume, reduced need for transfusion, and lower rate of ventricular fibrillation after ACC, as well as its cost-effectiveness and logistical convenience. In complex surgeries with a predicted ACC duration > 180 min, HTK cardioplegia offers a more potent myocardial protection strategy with the advantages of a more stable hemodynamic profile, lower TnI levels, and reduced need for IABP in prolonged ischemia. Tailoring clinical decision algorithms based on the predictive power of cardioplegic agents for complications, and not just their theoretical duration of action, may improve patient safety and surgical success.

## Figures and Tables

**Table 1:** Propensity comparison of basic demographic and clinical variables before and after propensity score matching (age, gender, BMI, preoperative EF, and type of surgery).

			Unmatched								Matched							
			DN Group (*n* = 384)			HTK Group (*n* = 298)			*p*	SMD	DN Group (*n* = 160)			HTK Group (*n* = 160)			*p*	SMD
			Mean ± Sd		Median	Mean ± Sd		Median			Mean ± Sd		Median	Mean ± Sd		Median		
Age			61.08 ± 11.59		56.82	56.08 ± 10.26		57.00	0.04	0.46	55.01 ± 11.82		56.00	56.77 ± 9.64		57.00	0.211	0.16
Gender		Male	208	54.17%		196.00	65.77%		0.81	0.30	94	58.75%		84	52.50%		0.211	0.13
		Female	276	71.88%		141.00	47.32%		0.30	66	41.25%			76	47.50%		0.261	0.13
BMI			33.89 ± 4.38		27.12	27.02 ± 4.25		27.58	0.36	1.59	27.45 ± 4.32		26.96	28.01 ± 4.62		27.07	0.425	0.12
EF (preoperative)			54.30 ±7.39		51.21	50.30 ± 8.35		50.00	0.04	0.51	49.46 ± 7.54		50.00	48.51 ± 8.06		48.98	0.186	0.12
Surgical procedure																		
	CABG	*n*/%	147	38.28%		68	22.82%		0.01	0.23	25	15.63%		26	16.25%		0.661	0.02
	AVR	*n*/%	40	10.42%		14	4.70%		0.00	0.17	18	11.25%		19	11.88%		0.741	0.02
	MVR	*n*/%	55	14.32%		19	6.38%		0.00	0.21	31	19.38%		29	18.13%		0.402	0.03
	AVR+MVR+TrR	*n*/%	10	2.60%		27	9.06%		0.48	0.27	17	10.63%		18	11.25%		0.489	0.02
	AVR+MVR	*n*/%	34	8.85%		44	14.77%		0.02	0.20	5	3.13%		3	1.88%		0.474	0.08
	MVR+TrR	*n*/%	19	4.95%		37	12.42%		0.26	0.27	11	6.88%		10	6.25%		0.516	0.03
	Valve Surgery+CABG	*n*/%	36	9.38%		27	9.06%		0.40	0.02	16	10.00%		18	11.25%		0.330	0.04
	Aort surgery	*n*/%	38	9.90%		56	18.79%		0.01	0.27	34	21.25%		33	20.63%		0.330	0.02
	Others	*n*/%	5	1.30%		6	2.01%		0.12	0.06	3	1.88%		4	2.50%		0.330	0.04

Data are presented as the mean ± standard deviation, median (IQR), or *n* (%). *p*-values indicate comparisons between groups. Standardized mean difference (SMD) values were calculated to assess the balance between groups after PSM. Variables with SMD < 0.1 indicate a significant balance between the two groups. DN, Del Nido; HTK, histidine-tryptophan-ketoglutarate; Sd, standard deviation; BMI, body mass index; EF, ejection fraction; CABG, coronary artery bypass graft; AVR, aortic valve replacement; MVR, mitral valve replacement; and TrR, tricuspid ring annuloplasty.

**Table 2:** Baseline patient characteristics and demographic data.

	DN Cardioplegia			HTK Cardioplegia			*p*
	Mean ± SD		Median	Mean ± SD		Median	
Female gender (*n*/%)	66	41.25%		76	47.50%		0.261
Age	55.01 ± 11.82		56.00	56.77 ± 9.64		57.00	0.211
Weight (kg)	77.77 ± 12.85		77.00	78.45 ± 13.32		77.00	0.615
BMI	27.45 ± 4.32		26.96	28.01 ± 4.62		27.07	0.425
EF (preoperative) %	49.46 ± 7.54		50.00	48.51 ± 8.06		48.98	0.186
	*n*	%		*n*	%		
Previous cardiac surgery	5	3.13%		3	1.88%		0.474
HT	27	16.88%		30	18.75%		0.661
DM	20	12.50%		22	13.75%		0.741
HL	35	21.88%		29	18.13%		0.402
PAD	17	10.63%		21	13.13%		0.489
Emergency surgery	2	1.25%		2	1.25%		0.975
COPD	13	8.13%		10	6.25%		0.516
Current smoker	19	11.88%		25	15.63%		0.330
AF preoperative	24	15.00%		19	11.88%		0.412

DN, Del Nido; HTK, histidine-tryptophan-ketoglutarate; SD, standard deviation; BMI, body mass index; HT, hypertension; EF, ejection fraction; DM, diabetes mellitus; HL, hyperlipidemia; PAD, peripheral artery disease; COPD, chronic obstructive pulmonary disease; and AF, atrial fibrillation.

**Table 3:** Perioperative data and procedural data of the DN group and the HTK group.

Variable	DN Cardioplegia (*n*:160)			HTK Cardioplegia (*n*:160)			*p*
	Mean ± SD		Median	Mean ± SD		Median	
Intraoperative Data							
Total CPB time (min)	196.04 ± 30.97		188.00	201.86 ± 38.418		200.00	0.061
ACC time (min)	145.80 ± 33.26		145.50	150.64 ± 35.997		145.50	0.317
Temperature (°C)	30.69 ± 5.59		31.00	30.12 ± 1.768		30.00	0.141
Amount of AC (mL)	1028.75 ± 322.02		1000.00	1417.81 ± 362.397		1500.00	<0.001
Cardioplegia initial dose (mL)	1250.63 ± 254.77		1200.00	1695.63 ± 328.585		1550.00	<0.001
Amount total cardioplegia (mL)	2039.38 ± 528.48		2100.00	1722.19 ± 317.456		2000.00	<0.001
	*n*	%		*n*	%		
Defibrillation requirement	6	3.75%		15	9.68%		0.042
Need for pace	2	1.25%		4	2.58%		0.388
Cardioplegia Delivery Method							
AC	76	47.50%		67	41.88%		0.380
ARC	84	52.50%		93	58.13%		0.380

CPB, cardiopulmonary bypass; ACC, aortic cross-clamp; AC, antegrade cardioplegia; ARC, antegrade and retrograde cardioplegia.

**Table 4:** Early postoperative and clinical outcomes of cardiac surgery.

Postoperative Variables		Del Nido Cardioplegia			HTK Cardioplegia			*p*
		Mean ± Sd		Median	Mean ± Sd		Median	
	Mean ICU stay (h)	39.90 ± 23.79		24.00	39.75 ± 23.04		24.00	0.976
	Intubation time (h)	10.70 ± 9.31		8.00	11.24 ± 12.30		8.00	0.654
	Mean hospital stay (day)	8.51 ± 2.32		8.00	9.09 ± 2.99		9.00	0.182
	Amount of drainage(mL)	684.38 ± 240.03		650.00	661.25 ± 240.28		600.00	0.319
Blood Products								
	Transfused ES (unit)	1.89 ± 0.96		2.00	2.21 ± 1.18		2.00	0.004
	Transfused FFP (unit)	2.80 ± 1.30		3.00	2.84 ± 1.14		3.00	0.830
	Transfused TS (unit)	0.49 ± 1.36		0.00	0.33 ± 1.07		0.00	0.259
Inotrope Score								
	İnotrope Score	14.13 ± 10.79		12.50	12.86 ± 10.87		10.00	0.189
EF (postoperative)		44.60 ± 8.00		45.00	44.65 ± 8.03		45.00	0.82
		*n*	%		*n*	%		
Mechanical Cardiac Support								
	IABP requirement	13	8.13%		11	6.88%		0.671
	ECMO requirement	2	1.25%		5	3.13%		0.252
Postoperative Complications						0.00%		
	Renal injury	16	10.00%		21	13.13%		0.729
	Dialysis	5	3.13%		4	2.50%		0.735
	Acute liver injury	5	3.13%		9	5.63%		0.274
	Stroke	5	3.13%		3	1.88%		0.474
	MODS	4	2.50%		5	3.13%		0.735
	Pneumonia	10	6.25%		11	6.88%		0.821
	Wound infection	4	2.50%		5	3.13%		0.735
	Re-exploration for bleeding	2	1.25%		4	2.50%		0.410
	Re-intubation (respiratory causes)	5	3.13%		7	4.38%		0.556
	Re-hospitalization	8	5.00%		5	3.13%		0.396
	AF	20	12.50%		24	15.00%		0.516
	LCOS	20	12.50%		11	6.88%		0.089
	Mortality	8	5.00%		7	4.38%		0.791

DN, Del Nido; HTK, histidine-tryptophan-ketoglutarate; LCOS, low cardiac output syndrome; AF, atrial fibrillation; EF, ejection fraction; ICU, intensive care unit; MODS, multiple organ dysfunction syndrome; TS: thrombocyte suspension; ES: erythrocyte suspension; FFP: fresh frozen plasma; IABP, intra-aortic balloon pump; and ECMO, extracorporeal membrane oxygenation.

**Table 5:** Postoperative biochemical analyses and myocardial damage markers.

	Del Nido Cardioplegia		HTK Cardioplegia		*p*
	Mean ± Sd	Median	Mean ± Sd	Median	
PH					
pre-CPB	7.39 ± 0.08	7.40	7.41 ± 0.07	7.42	0.138
post-CPB	7.39 ± 0.09	7.40	7.40 ± 0.07	7.40	0.974
Lactate (mmol/L)					
pre-CBP	3.03 ± 1.72	2.65	3.20 ± 1.84	2.80	0.183
post-CBP	4.63 ± 2.17	4.39	4.38 ± 2.18	3.91	0.216
Na (mmol/L)					
Preoperative	139.73 ± 3.23	140.00	139.79 ± 2.63	140.00	0.736
Postoperative	135.51 ± 5.16	136.00	134.02 ± 3.80	134.00	0.003
K^+^ (mmol/L)					
pre-CBP	4.51 ± 0.80	4.45	4.50 ± 0.74	4.40	0.947
post-CBP	4.65 ± 0.72	4.60	4.71 ± 0.69	4.60	0.526
Urea (mg/dL)					
Preoperative	36.06 ± 9.90	37.80	34.99 ± 8.32	35.05	0.279
Postoperative	43.16 ± 10.57	41.15	43.16 ± 11.09	41.45	0.713
Creatinine (mg/dL)					
Preoperative	1.58 ± 6.96	1.04	1.14 ± 1.80	1.00	0.323
Postoperative	1.36 ± 0.60	1.23	1.39 ± 0.55	1.23	0.592
Hematocrit (%)					
Preoperative	36.88 ± 3.10	37.01	36.68 ± 3.43	36.89	0.570
Postoperative	26.66 ± 2.14	26.81	25.83 ± 2.05	25.95	0.000
WBC (/µL)					
Preoperative	9.66 ± 3.17	9.66	9.16 ± 2.93	9.45	0.241
Postoperative	12.42 ± 3.90	11.69	12.56 ± 3.65	12.12	0.478
Platelets (/µL)					
Preoperative	194.43 ± 71.65	189.00	205.72 ± 75.87	209.00	0.177
Postoperative	152.78 ± 43.01	143.50	145.03 ± 36.56	138.00	0.174
CRP (mg/L)					
CRP pre-CBP	4.83 ± 7.71	0.40	5.55 ± 9.12	0.40	0.434
CRP post-CRP	33.55 ± 55.46	8.25	21.79 ± 33.04	1.75	0.240
LDH (U/Lt)					
Preoperative	286.50 ± 94.25	264.00	276.87 ± 84.98	259.00	0.415
Postoperative	401.95 ± 130.74	386.50	396.96 ± 144.11	366.50	0.441
AST (U/Lt)					
Preoperative	28.44 ± 11.96	27.50	27.87 ± 10.67	27.00	0.687
Postoperative	47.30 ± 111.28	32.00	58.32 ± 144.43	29.00	0.356
ALT (U/lt)					
Preoperative	33.07 ± 10.60	33.50	34.17 ± 15.78	34.00	0.672
Postoperative	54.95 ± 113.64	38.00	81.25 ± 205.35	40.00	0.428
CK-MB (µg/L)					
Preoperative	2.68 ± 1.41	2.80	2.70 ± 1.34	2.78	0.960
Postoperative (4 h)	29.82 ± 9.41	28.82	29.65 ± 8.66	28.38	0.897
Postoperative (24 h)	38.85 ± 16.61	35.80	39.80 ± 17.61	36.04	0.891
Troponin (ng/L)					
Preoperative	0.68 ± 0.29	0.71	0.68 ± 0.31	0.67	0.781
Postoperative (4 h)	6.55 ± 4.10	5.87	5.58 ± 2.21	5.14	0.003
Postoperative (24 h)	9.84 ± 7.20	8.26	8.83 ± 3.97	8.10	0.151

CPB, cardiopulmonary bypass; WBC, white blood cell; CRP, C-reactive protein; LDH, lactate dehydrogenase; AST, aspartate aminotransferase; ALT, alanine aminotransferase; and CK-MB, creatine kinase MB isotype.

**Table 6:** Subgroup analyses of DN and HTK cardioplegic solutions in terms of adequacy and safety of myocardial protection according to ACC duration (Group A: 90–120 min, Group B: 120–150 min, Group C: 150–180 min, Group D: >180 min.).

		Del Nido Cardioplegia		HTK Cardioplegia		*p*
		Mean ± Sd	Median	Mean ± Sd	Median	
Group A (ACC time: 90–120 min) (*n*/%)		45 (57.69%)		33 (42.31%)		
	EF (postoperative)	47.56 ± 6.08	50.00	49.18 ± 8.19	50.00	0.907
	Inotrope score	12.93 ± 9.67	10.00	14.70 ± 12.28	10.00	0.737
	Lactate (mmol/L)	3.71 ± 1.62	3.40	3.68 ± 2.33	3.10	0.430
	LDH (U/Lt)	383.73 ± 139.42	374.00	400.36 ± 127.84	364.00	0.537
	CK-MB (4 h) ng/mL	27.91 ± 6.61	27.74	28.16 ± 7.67	27.30	0.606
	CK-MB (24 h) ng/mL	41.04 ± 24.40	32.28	39.84 ± 20.84		0.682
	Tn I (4 h) ng/mL	5.21 ± 1.41	4.80	5.74 ± 2.63	5.04	0.541
	Tn I (24 h) ng/mL	9.52 ± 4.31	8.22	8.81 ± 4.10	7.03	0.424
	Mean ICU stay (h)	41.60 ± 32.32	24.00	44.73 ± 30.78	24.00	0.533
	Mean hospital stay(h)	8.89 ± 2.30	9.00	9.06 ± 2.65	8.00	0.988
	Defibrillation requirement (*n*/%)	2	1.25%	3	1.94%	0.408
	IABP requirement (*n*/%)	1	0.63%	1	0.65%	1.000
	ECMO requirement (*n*/%)	0	0.00%	1	0.65%	0.423
	LCOS (*n*/%)	1	0.63%	0	0.00%	1.000
	Mortality (*n*/%)	0	0.00%	1	0.65%	0.423
Group B (ACC time: 120–150 min) (*n*/%)		43 (52.44%)		39 (47.56%)		
	EF (postoperative)	47.35 ± 8.44	47.00	46.26 ± 6.11	45.00	0.524
	Inotrope score	12.33 ± 10.45	8.00	11.26 ± 8.05	10.00	0.744
	Lactate (mmol/L)	3.61 ± 1.66	3.30	3.73 ± 2.11	3.10	0.956
	LDH (U/Lt)	421.70 ± 135.41	390.00	398.26 ± 109.89	374.00	0.480
	CK-MB (4 h) ng/mL	31.12 ± 18.07	28.41	29.73 ± 5.64	30.00	0.629
	CK-MB (24 h) ng/mL	50.57 ± 65.07	34.94	41.24 ± 18.94	36.20	0.937
	Tn I (4 h) (ng/mL)	5.77 ± 1.84	5.87	5.52 ± 1.78	5.27	0.510
	Tn I (24 h (ng/mL)	9.32 ± 4.18	7.66	10.03 ± 5.29	8.30	0.639
	Mean ICU stay (h)	36.84 ± 15.36	36.00	38.77 ± 20.87	24.00	0.931
	Mean hospital stay(h)	7.88 ± 2.23	7.00	8.69 ± 2.26	9.00	0.056
	Defibrillation requirement (*n*/%)	1	0.63%	4.00	0.03	0.134
	IABP requirement (*n*/%)	1	0.63%	3.00	0.02	0.342
	ECMO requirement (*n*/%)	1	0.63%	2.00	0.01	0.652
	LCOS (*n*/%)	3	1.88%	2.00	0.01	0.727
	Mortality (*n*/%)	2	1.25%	2.00	0.01	1.000
Group C (ACC time: 150–180 min) (*n*/%)		39 (49.37)		40 (50.63%)		
	EF (postoperative)	44.67 ± 8.57	45.00	44.13 ± 6.49	42.50	0.207
	Inotrope score	11.59 ± 6.97	10.00	14.40 ± 11.51	13.00	0.518
	Lactate (mmol/L)	5.16 ± 2.20	4.93	4.11 ± 1.56	4.10	0.025
	LDH (U/Lt)	372.31 ± 113.27	352.00	370.20 ± 160.89	333.00	0.524
	CK-MB (4 h) ng/mL	28.96 ± 6.42	29.67	32.23 ± 16.83	31.07	0.550
	CK-MB (24 h)	41.68 ± 29.50	31.73	45.74 ± 59.08	29.69	0.645
	Tn I (4 h) (ng/mL)	6.35 ± 2.34	5.72	6.41 ± 2.86	6.03	0.945
	Tn I (24 h) (ng/mL)	8.91 ± 3.66	8.19	10.32 ± 4.68	9.44	0.134
	Mean ICU stay (h)	37.23 ± 19.43	24.00	36.60 ± 22.07	24.00	0.809
	Mean hospital stay(h)	8.90 ± 2.55	8.00	9.15 ± 3.68	9.00	0.774
	Defibrillation requirement (*n*/%)	2	1.25%	5.00	0.03	0.249
	IABP requirement (*n*/%)	5	3.13%	2.00	0.01	0.221
	ECMO requirement (*n*/%)	1	0.63%	0.00	0.00	0.494
	LCOS (*n*/%)	1	0.63%	1.00	0.01	1.000
	Mortality (*n*/%)	2	1.25%	1.00	0.01	0.615
Group D (ACC time > 180 min) (*n*/%)		33 (43.42%)		43 (56.58%)		
	EF (postoperative)	39.00 ± 6.01	40.00	41.63 ± 7.29	40.00	0.092
	Inotrope score	14.64 ± 12.74	8.00	14.12 ± 13.77	10.00	0.701
	Lactate (mmol/L)	6.58 ± 1.90	6.46	5.52 ± 2.21	5.12	0.008
	LDH (U/Lt)	436.09 ± 125.05	432.00	420.49 ± 172.52	376.00	0.118
	CK-MB (4 h) ng/mL	38.66 ± 14.61	34.16	35.35 ± 17.29	26.65	0.105
	CK-MB (24 h)	51.05 ± 27.43	43.24	41.36 ± 49.97	30.24	0.077
	Tn I (4 h) (ng/mL)	6.87 ± 2.10	6.98	4.95 ± 1.91	4.46	0.000
	Tn I (24 h) (ng/mL)	10.52 ± 4.65	9.15	9.17 ± 4.23	8.14	0.123
	Mean ICU stay (h)	44.73 ± 23.86	48.00	40.19 ± 19.92	36.00	0.445
	Mean hospital stay(h)	8.33 ± 2.09	9.00	9.67 ± 3.14	9.00	0.108
	Defibrillation requirement (*n*/%)	1	0.63%	3	1.94%	0.628
	IABP requirement (*n*/%)	8	5.00%	3	1.94%	0.034
	ECMO requirement (*n*/%)	0	0.00%	2	1.29%	0.502
	LCOS (*n*/%)	5	3.13%	2	1.29%	0.117
	Mortality (*n*/%)	4	2.50%	3	1.94%	0.442

EF, ejection fraction; LDH, lactate dehydrogenase; troponin I, Tn I; CK-MB, creatine kinase MB isotype; ICU, intensive care unit; IABP, intra-aortic balloon counter pulsation; ECMO, extracorporeal membrane oxygenation; and LCOS, low cardiac output syndrome.

**Table 7:** Comparison of ROC analysis results for predicting major postoperative outcomes based on aortic cross-clamp time in Del Nido and HTK cardioplegia groups.

Variables	DN Cardioplegia							HTK Cardioplegia						
	AUC	*p*	95% CI		Cut-Off	Sensitivity	Specificity	AUC	*p*	95% CI		Cut-Off	Sensitivity	Specificity
LCOS	0.746	0.000	0.633	0.86	161	70.00%	73.60%	0.696	0.074	0.551	0.842	204	36.00%	93.30%
VIS > 15	0.620	0.010	0.526	0.71	183.5	31.30%	95.80%	0.661	0.001	0.571	0.75	142.5	73.10%	55.60%
IABP requirement	0.804	0.000	0.689	0.919	168.500	84.60%	76.90%	0.630	0.150	0.482	0.779	180.50	54.50%	77.20%
Mortality	0.740	0.077	0.589	0.891	161.000	75.00%	70.40%	0.571	0.526	0.349	0.793	164.50	57.10%	65.40%

AUC, area under the curve; CI, confidence interval; LCOS, low cardiac output syndrome; VIS, vasoactive inotrope score; and IABP, intra-aortic balloon pump.

**Table 8:** Logistic regression results for predictors of major postoperative outcomes in DN and HTK groups.

		Univariate Model				Multivariate Model			
		OR	95% C.I		*p*	OR	95% C.I		*p*
Postoperative LCOS development									
	Age	0.978	0.946	1.011	0.182	0.960	0.924	0.998	0.041
	Gender	0.725	0.345	1.522	0.395				
	BMI	1.010	0.930	1.096	0.820				
	ACC Time (min)	1.024	1.013	1.036	<0.001	1.023	1.010	1.036	<0.001
	Type of Cardioplegia	0.517	0.239	1.117	0.093				
	Postoperative EF	0.907	0.860	0.957	<0.001	0.917	0.865	0.971	0.003
	Postoperative Troponin I (4 h)	1.109	1.000	1.230	0.050				
	Postoperative Troponin I (24 h)	1.150	1.059	1.249	<0.001	1.154	1.053	1.265	0.002
	Postoperative CK-MB (4 h)	1.035	0.997	1.074	0.074				
	Postoperative CK-MB (24 h)	1.017	0.998	1.035	0.080				
VIS > 15									
	Age	0.991	0.971	1.012	0.420				
	Gender	1.353	0.852	2.148	0.201				
	BMI	1.023	0.973	1.077	0.375				
	ACC Time (min)	0.015	1.008	1.022	<0.001	1.015	1.008	1023	<0.001
	Type of Cardioplegia	1.385	0.876	2.188	0.163	1.529	0.946	2.470	0.083
	Postoperative EF	0.963	0.935	0.992	0.013	0.965	0.936	0.995	0.021
	Postoperative Troponin I (4 h)	1.020	0.954	1.091	0.563				
	Postoperative Troponin I (24 h)	1.034	0.987	1.083	0.155				
	Postoperative CK-MB (4 h)	1.025	0.999	1.051	0.058				
	Postoperative CK-MB (24 h)	1.006	0.993	1.020	0.354				
IABP requirement									
	Age	0.464	0.950	1.024	0.986				
	Gender	1.127	0.485	2.619	0.781				
	BMI	1.017	0.929	1.115	0.710				
	ACC Time (min)	1.020	1.008	1.032	0.001	1.020	1.007	1.032	0.002
	Type of Cardioplegia	1.198	0.520	2.760	0.672				
	Postoperative EF	0.990	0.940	1.043	0.710				
	Postoperative Troponin I (4 h)	1.030	0.938	1.131	0.535				
	Postoperative Troponin I (24 h)	0.993	0.915	1.076	0.856				
	Postoperative CK-MB (4 h)	1.037	0.995	1.081	0.083				
	Postoperative CK-MB (24 h)	1.026	1.007	1.046	0.008	1.027	1.007	1.049	0.010
Postoperative mortality									
	Age	0.980	0.936	1.027	0.401				
	Gender	1.207	0.419	3.475	0.727				
	BMI	0.866	0.750	0.998	0.048	0.866	0.750	1.001	0.049
	ACC Time (min)	1.014	0.999	1.029	0.059	1.016	1.000	1.031	0.048
	Type of Cardioplegia	1.150	0.407	3.251	0.792				
	Postoperative EF	0.982	0.920	1.049	0.588				
	Postoperative Troponin I (4 h)	0.974	0.799	1.187	0.794				
	Postoperative Troponin I (24 h)	1.019	0.958	1.083	0.551				
	Postoperative CK-MB (4 h)	1.019	0.966	1.075	0.483				
	Postoperative CK-MB (24 h)	0.986	0.951	1.022	0.444				

ACC, aortic cross-clamp; BMI, body mass index; CI, confidence interval; CK-MB, creatine kinase-myocardial band; EF, ejection fraction; HTK, histidine-tryptophan-ketoglutarate; IABP, intra-aortic balloon pump; LCOS, low cardiac output syndrome; OR, odds ratio; and VIS, vasoactive inotrope score.

## Data Availability

Raw data supporting the conclusions of this study will be made available by the authors upon request.
